# Impact of an e-learning platform in improving dentists’ CBCT diagnostic skills: a pilot study

**DOI:** 10.1007/s11282-026-00954-2

**Published:** 2026-07-24

**Authors:** Daniel Brockes, Caroline V. Busch, Kathrin Becker, Jürgen Becker, Giulia Brunello, Katharina Mücke

**Affiliations:** 1 Department of Oral Surgery, University Hospital Düsseldorf, Moorenstr. 5, 40225 Düsseldorf, Germany; 2 Department of Oral Medicine, Infection, and Immunity, Harvard School of Dental Medicine, Boston, MA USA; 3 Department of Orthodontics and Dentofacial Orthopaedics, Charité, Universitätsmedizin, Freie Universität Berlin and Humboldt‐Universität zu Berlin, Aßmannshauser Str. 4-6, 14197 Berlin, Germany; 4 Department of Orthodontics, University Hospital Düsseldorf, Moorenstr. 5, 40225 Düsseldorf, Germany

**Keywords:** 3D imaging, Postgraduate education, Dentistry, Radiology

## Abstract

**Objectives:**

Cone Beam Computed Tomography (CBCT) is a widely used imaging technology in dentistry, requiring specialized training and a specific license in Germany. This study aimed to evaluate the effectiveness of an e-learning platform for acquiring CBCT knowledge and to examine participant satisfaction and experiences.

**Methods:**

The study included German dentists, half with CBCT license and half without. The participants completed an online pre-test with 15 image-based questions to assess basic knowledge and then were given access to the e-platform containing 104 annotated CBCT cases (e.g. implant planning, cysts, or impacted teeth). After 8–10 weeks, a post-test similar to the pre-test was conducted. An 11-item questionnaire recorded participant experience. Data were analysed using independent and paired *t*-tests, and Fisher’s exact test; *p* < 0.05 was considered statistically significant.

**Results:**

Between May and October 2025, 32 dentists participated in the study (16 with and 16 without CBCT license). Learning with the e-learning platform led to a significant increase in knowledge (*p* < 0.001). Participants with CBCT license had higher baseline scores than those without (75.8% vs. 69.3%; *p* < 0.05); after the learning phase, the difference was no longer significant (*p* = 0.37), with final scores of 83.1% and 80.6%, respectively. Regardless of CBCT license status, dentists found the platform to be a valuable tool for enhancing diagnostic skills, offering a user-friendly, case-based structure.

**Conclusions:**

The digital learning platform significantly improved CBCT-related diagnostic skills and dentists, both with and without a CBCT license, found the platform practical and easy to use.

**Supplementary Information:**

The online version contains supplementary material available at https://doi.org/10.1007/s11282-026-00954-2.

## Introduction

Cone-beam computed tomography (CBCT) is an increasingly valuable radiological tool in dental practice [1]. Unlike conventional two-dimensional (2D) radiographs, CBCT provides three-dimensional (3D) visualization of anatomical structures and pathologies [2], which enables improved diagnosis, treatment planning, and more predictable procedures across various dental specialties [3, 4].

CBCT plays an important role in modern surgical and prosthetic treatments, especially implant dentistry, where anatomical assessment is critical [5]. As surgical procedures, including implant treatments, become increasingly common and a growing number of general dentists pursue advanced training in this field, the demand for structured CBCT education continues to rise [3].

To use CBCT effectively, dental professionals need a solid understanding of anatomy, practical skills in 3D orientation in CBCT navigation, and specific radiological knowledge [6].

In Germany, the use of CBCT is regulated by strict legal frameworks, including the Radiation Protection Act (*Strahlenschutzgesetz*, StrlSchG) [7] and the Radiation Protection Ordinance (*Strahlenschutzverordnung*, StrlSchV) [8]. Dentists in Germany are only authorized to use ionizing radiation if they hold the appropriate radiation protection qualification (i.e., *Fachkunde im Strahlenschutz*) [9]. According to the official guidelines for radiation protection expertise in dentistry (i.e., *Fachkunderichtlinie Zahnmedizin*), a separate CBCT-specific qualification is required in Germany, which includes the successful completion of a certified training program consisting of two theoretical modules, a practical session with the evaluation of 25 CBCT cases, followed by a written examination. The certificate must be renewed every five years through refresher courses.

Despite different regulatory requirements, CBCT is widely used in daily dental practice not only in Germany but across Europe, and many clinicians who acquire or interpret CBCT data have received limited formal education in 3D imaging [10].

In this context, digital learning platforms are gaining increasing importance in dental education, particularly in image-based disciplines such as oral and maxillofacial radiology [11–13]. A longitudinal study from our group demonstrated that an interactive Oral Radiology Platform (ORP) containing 2D radiographic images significantly improved undergraduate students’ diagnostic skills in oral radiology [14]. The study included various online learning modules and showed that such digital formats could be as effective, or even more effective, than traditional learning approaches alone.

Based on these positive results of Mücke et al. [14], the ORP has now been extended to include CBCT-related content, using annotated 2D screenshots derived from 3D datasets. This extension is particularly relevant for postgraduate education, as it allows clinicians to explore a wide range of CBCT cases within a digital learning environment. Indeed, despite the growing integration of CBCT technologies into routine clinical practice, a significant gap remains in the literature regarding the effectiveness of e-learning platforms in supporting postgraduate education.

To evaluate the effectiveness of this CBCT-implanted platform, German dentists were given access to the platform, and knowledge gain was measured between baseline and 8–10 weeks after the first access. The primary aim of the study was to determine whether access to the ORP improved dental professionals’ competencies to interpret CBCT images. The secondary aim was to investigate whether baseline knowledge and improvements differed between participants with and without an official German CBCT license. Finally, the study aimed to explore the perceptions of the participants of the ORP, including whether they perceived a knowledge gain and whether learning with platform had an impact on their clinical usage of CBCT data.

## Materials and methods

The study protocol was submitted to and approved by the Ethics Committee of the University of Dusseldorf (protocol no. 2024-2977).

### Study design and population

This prospective longitudinal observational pilot study used a pre-test/post-test design. Participants were dentists holding a German dental license who had been working in clinical practice for at least six months.

To recruit an equal number of participants with and without a CBCT license, individuals were asked, before starting the pre-test, whether they already held a license or planned to obtain one soon. This approach allowed us to identify and exclude participants who initially reported not having a license but planned to obtain one before the post-test, as they might have received additional CBCT training outside the study.

Participants had 8 to 10 weeks to use the ORP for individual learning and preparation for the post-test. Access was provided via the SmartZoom® platform (Smart In Media AG, Cologne, Germany) and could be used at any time with an internet connection on a tablet, laptop, or PC. The pre- and post-tests, as well as the ORP, were provided entirely in German.

Data collection took place, between 25th May 2025 and 10th October 2025, at the Dental Clinic of the University Hospital Düsseldorf. Before the pre-test, participants received a written information sheet describing the study protocol, approved by the local ethics committee, and outlining their rights regarding data protection, including the option to withdraw from the study and request deletion of their data at any time. Written informed consent was obtained from all participants before the study began.

In both the pre- and post-test, participants were first asked to provide their names to enable data matching. All data were then pseudonymized to ensure participant confidentiality.

Participants received a personalized link granting access to the test on the SurveyMonkey® online survey platform (SurveyMonkey Inc., San Mateo, CA, USA). All tests were carried out under supervision to prevent the use of external resources. The tests were displayed on identical computer monitors, and participants were able to zoom in on the CBCT images if needed, ensuring that all relevant image details could be assessed. Participants received their personal login credentials via email immediately after the pre-test to get access to the *Oral Radiology Platform*. Participant compliance with the e-learning program was encouraged through monthly email reminders sent throughout the study period. Access to the platform was provided and verified for all participants. Detailed usage data, such as total time spent on the platform or completion of individual learning modules, were not available and therefore could not be assessed.

After completing the post-test, participants were given a paper-based evaluation form to capture their subjective experience with the platform.

### Oral radiology platform

The home page of the ORP presented a short description of the tool, its content, and its educational aims. It comprised two main sections: “Planes, Devices, and Anatomy in CBCT” and “2D Screenshots”.

**Fig. 1:**
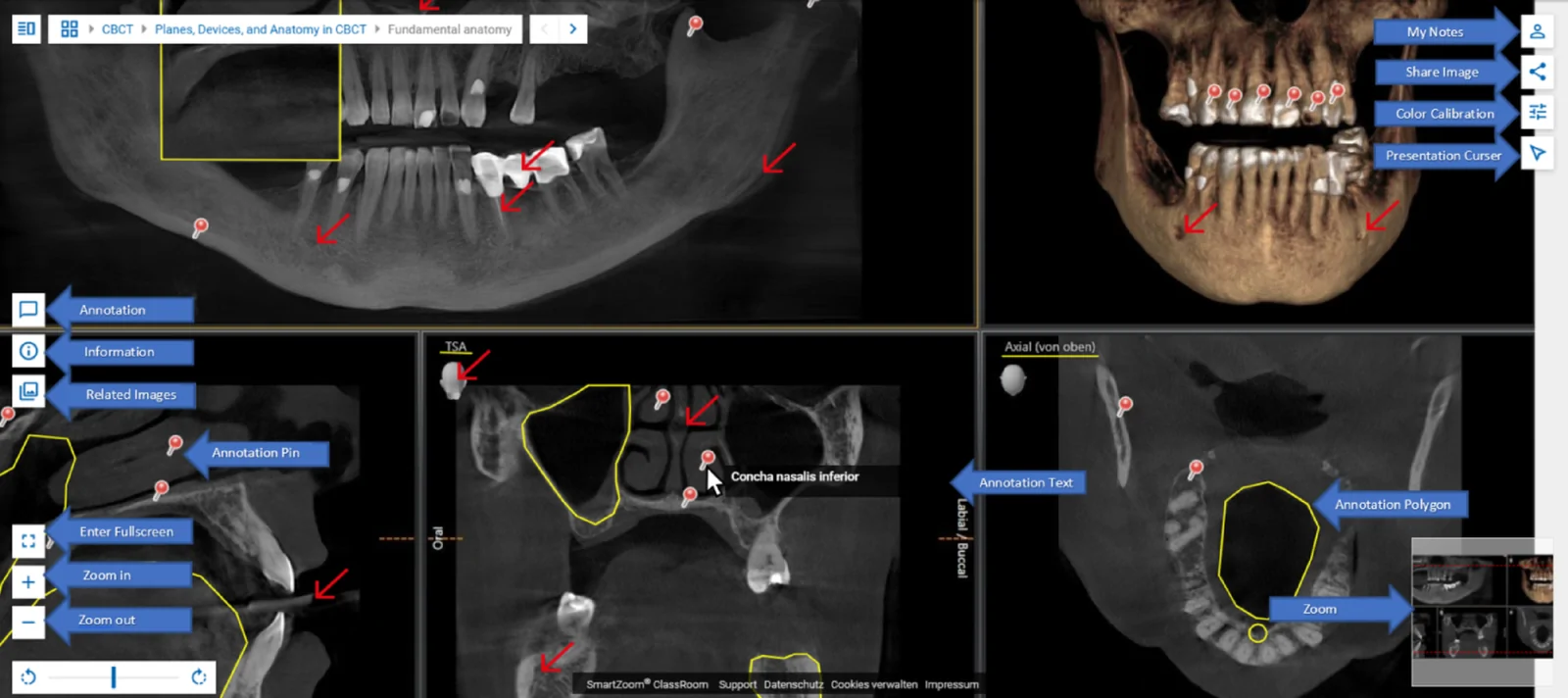
Representative annotated case with text translated into English belonging to the “Planes, Devices, and Anatomy in CBCT” section, showing fundamental anatomy. Interactive options available to the user included adding notes, adjusting contrast (e.g., brightness), and accessing a general information folder with explanations of the findings and CBCT advantages. Additional tools include zoom, rotation, full-screen view, and a presentation cursor

**Fig. 2:**
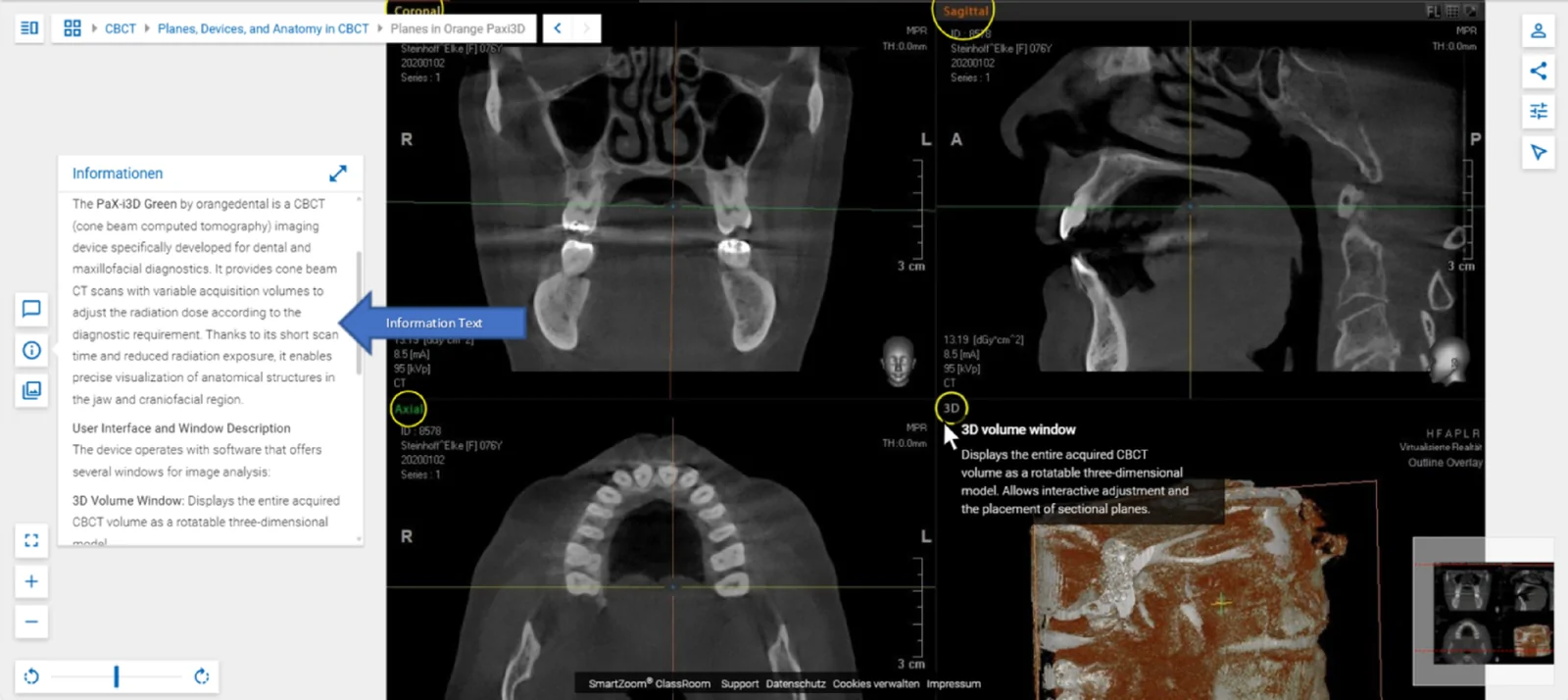
Representative annotated case with text translated into English belonging to the “Planes, Devices, and Anatomy in CBCT” section, showing an example case from Orange Pax-i3D Green (orangedental GmbH & Co.KG, Biberach an der Riß, Germany). On the left side of the interface, the open folder displays the information text

The first section provided an overview of how the data was displayed in the software associated with the CBCT devices from which the radiological datasets had been acquired, i.e., Romexis for Planmeca Promax device (Planmeca, Helsinki, Finland), Sidexis 4 for Sirona Orthophos SL (Dentsply Sirona Inc., Charlotte, NC, USA), and Ez3D Plus for Orange Pax-i3D Green (orangedental GmbH & Co.KG, Biberach an der Riß, Germany). For each device, an example image was provided together with annotations highlighting the main technical settings. These also contained annotations demonstrating how different imaging planes correspond to patient positioning and how to navigate these views when answering clinical questions (Fig. 1). In addition, a CBCT scan was included in this section to showcase the most important anatomical structures, such as the tongue, nasal cavity, and ethmoidal sinus, serving as a reference example (Fig. 2).

**Fig. 3:**
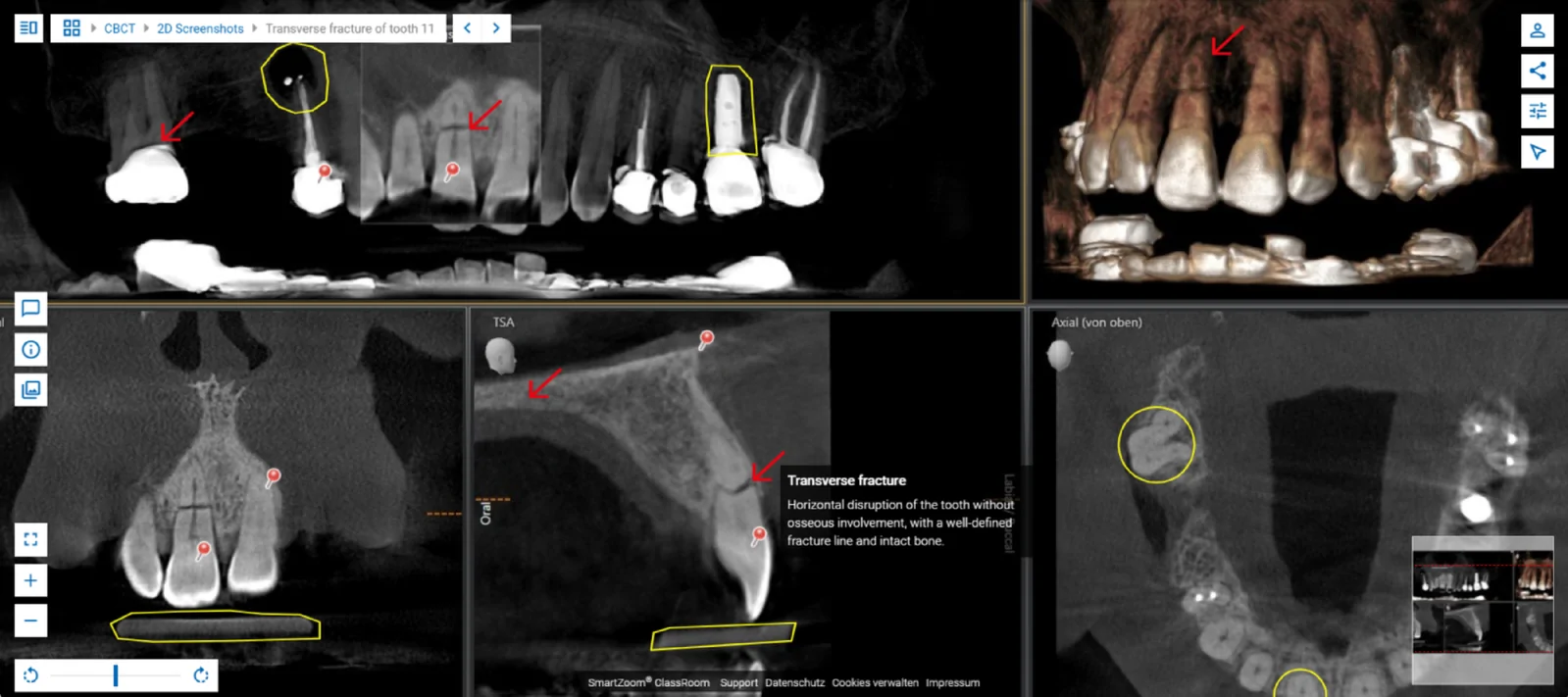
Representative annotated case with text translated into English belonging to the “2D Screenshots” section

The “2D Screenshots” section contained 97 annotated CBCT images (Fig. 3). These included a wide range of cases, such as teeth following endodontic treatment, anatomical variations, pathologies (e.g., cysts and tumours), and impacted teeth or orthodontic appliances visible in CBCT scans (Table 1). Furthermore, several examples illustrated image-quality issues, such as motion artefacts, helping learners to differentiate between technical limitations and true pathologies.

**Table 1:** Distribution and focus of the 97 annotated CBCT cases included in the “2D Screenshots” section of the oral radiology platform

Category	Description of content	Number of cases
Anatomical variations	Anatomical variations, key landmarks, and CBCT-relevant structures essential for interpretation	6
Oral surgery and implant dentistry	Surgical planning cases (e.g., implant sites, bone morphology) with emphasis on critical anatomical considerations	19
Oral Pathology	Cysts, tumours, and other lesions, each verified against corresponding histological diagnoses	54
Endodontics	Teeth after endodontic treatment, demonstrating post-treatment anatomy and radiological appearance	10
Orthodontics	Impacted teeth and orthodontic appliances, illustrating typical orthodontic findings in CBCT	8

All pathological cases included in the atlas had been checked against the corresponding histological diagnosis. This ensured that the CBCT findings presented to participants were consistent with definitive histological outcomes, when required.

### Knowledge gain

Thirty CBCT-based questions were developed in advance and finalized before data collection began. To ensure comparable difficulty between the two tests, the questions were ordered and then systematically divided: every second question was assigned to the pre-test, and the remaining questions to the post-test. This procedure avoided potential bias that might have arisen if the first 15 or last 15 questions had been grouped together. Each item was reviewed and validated by a panel of three dentists (C.V.B., G.B., and K.M.) with more than five years of clinical experience in CBCT diagnostics.

The pre-test consisted of 17 items and was structured as follows: the first item recorded the participant’s name, and the second determined whether they held an official CBCT license. The remaining 15 items were a mix of multiple-choice, single-choice, and Kprim-choice questions covering the different categories of the ORP (Table 1). Sample questions are provided in Online Appendix 1.

The post-test consisted of 16 items: the first recorded the participant’s name, followed by 15 CBCT-based items using new images.

Ninety seconds were allocated for each item. Therefore, the maximum duration was 25 min and 30 s for pre-test and 24 min for post-test.

### Psychometric analyses

Psychometric analyses within each test were performed to evaluate the reliability and validity of the tests assessing knowledge gain. Psychometric properties of the baseline and final examinations were examined separately.

Internal consistency was evaluated using Cronbach’s alpha using the following threshold values: ≥ 0.90 excellent internal consistency, 0.80–0.89 good internal consistency, 0.70–0.79 acceptable internal consistency, 0.60–0.69 questionable internal consistency, 0.50–0.59 poor internal consistency and < 0.50 unacceptable internal consistency [15].

Item discrimination was evaluated using corrected item-total correlations (r_it) [16], with ≥ 0.30 considered good discrimination, 0.20–0.29 acceptable discrimination, and < 0.20 poor discrimination.

Item difficulty was assessed considering the following threshold: 0.85–1 easy, 0.65–0.849 moderate, 0.45–0.649 moderately difficult, 0.25–0.449 difficult and 0–0.249 very difficult.

### Experience with the CBCT oral radiology platform

The evaluation questionnaire consisted of 11 questions, each offering the following response options: "Strongly agree", "Agree", "Disagree", "Strongly disagree", and "Not applicable". The evaluation was not time-limited to avoid any stress or time pressure during answering. The main aim of this evaluation was to assess overall satisfaction with the digital learning tool, particularly regarding its quality, usability, and perceived educational value.

The questions were grouped into two thematic categories:

- impact and motivation (7 items);
- usability and structure (4 items).

### Statistical analysis

For this pilot study, a convenience sample of at least 15 dentists per group was chosen, accounting for a potential dropout rate of 20%. This sample size is consistent with comparable studies examining digital radiology education formats in dentistry [12, 17].

Descriptive statistics (means, standard deviations) were calculated for the pre- and post-test, overall and within each group (with and without CBCT license). Normal distribution was assessed visually using boxplots.

To evaluate the overall knowledge gain, pre- and post-test scores of the same participants were compared using a paired* t*-test. Differences between participants with and without a CBCT license were analysed using an independent samples* t*-test for both pre- and post-test scores.

Responses from the evaluation questionnaire were analysed and displayed using bar graphs. Fisher's exact test was used to assess the influence of a CBCT license on platform evaluations. In the first analysis, "Strongly agree" and "Agree" were combined, as well as were "Disagree" and "Strongly disagree," with "Not applicable" excluded. A second analysis included "Not applicable" as a separate category. The data analysis was performed using the open-source software R [18] and the online survey tool SurveyMonkey® for data collection and export. The level of significance was set at *p* < 0.05.

## Results

All 32 participants, 16 with and 16 without CBCT license, completed both the pre- and post-test, resulting in no dropouts and a response rate of 100%. A checklist assessing adherence to the "Good Practice in the Conduct and Reporting of Survey Research" criteria [19] is provided in Online Appendix 2.

### Knowledge gain

Pre- and post-test results, overall and by group, are reported in Table 2.

**Table 2:** Pre- and post-test scores

Group	Timepoint	Mean (%)	SD (%)	Median (%)	IQR (%)	95% CI
Overall	Pre-test	72.52	9.17	73.00	68.92; 77.50	69.21; 75.83
	Post-test	81.84	7.71	84.00	76.15; 88.25	79.06; 84.63
With CBCT license	Pre-test	75.79	5.24	75.17	71.67; 81.33	73.00; 78.59
	Post-test	83.08	8.72	87.83	75.17; 89.00	78.44; 87.73
Without CBCT license	Pre-test	69.25	11.12	70.50	65.83; 76.50	63.32; 75.18
	Post-test	80.60	6.62	81.00	77.42; 84.33	77.08; 84.13

Data are expressed in %

**Fig. 4:**
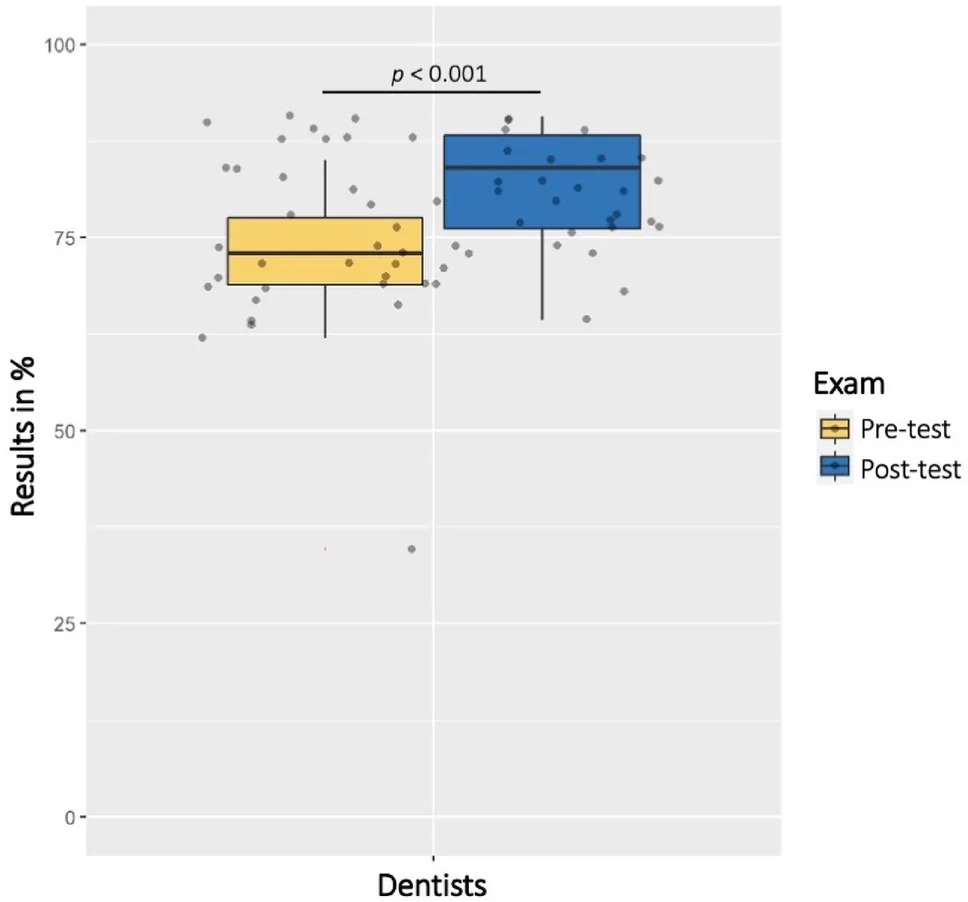
Boxplots showing the distribution of pre- and post-test scores for all tested dentists. The pre-test data is shown in yellow, while the post-test data is represented in blue

Participants showed a significant increase in CBCT interpretation scores. Mean performance improved from 72.52% in the pre-test to 81.84% in the post-test (*p* < 0.001) (Fig. 4), with a mean difference of 9.32%. The improvement was significant in the group with a CBCT license (*p* = 0.019), as well as in the group without (*p* = 0.002).

**Fig. 5:**
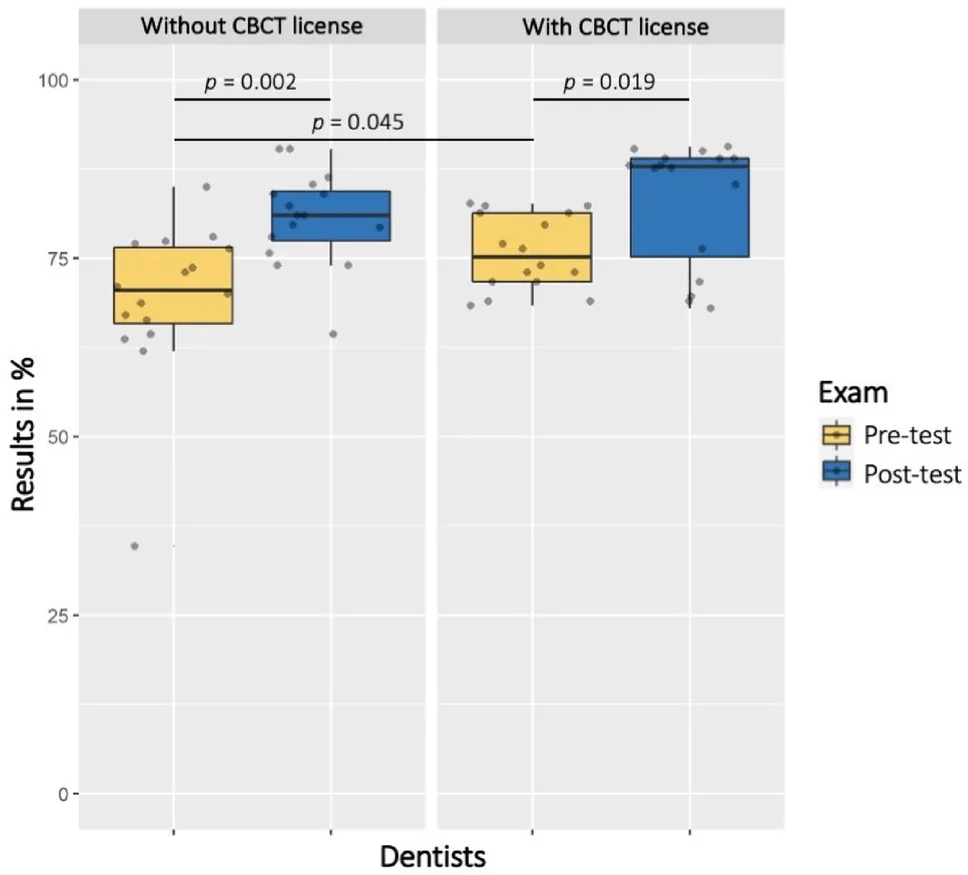
Boxplots showing the distribution of pre- and post-test scores in dentists with and without CBCT license. The pre-test data is shown in yellow, while the post-test data is represented in blue

Comparing participants by CBCT license status (Fig. 5), dentists with a CBCT license obtained significantly higher scores at baseline (75.79%) than participants without (69.25%) (*p* = 0.045). While no significant difference between groups was observed in post-test scores (*p* = 0.373), with 83.08% and 80.60% in dentists with and without a license, respectively (Fig. 5).

### Psychometric analyses

Internal consistency, assessed using Cronbach’s alpha, was questionable for the baseline assessment (*α* = 0.66) and poor for the final assessment (*α* = 0.54), indicating limited reliability for both tests.

Item discrimination was evaluated using corrected item-total correlations (r_it). At baseline, most items (9/15) demonstrated acceptable discrimination (r_it ≥ 0.30), indicating adequate differentiation between higher- and lower-performing participants. In contrast, the final exam showed weaker item discrimination, with several items falling below the recommended threshold (10/15), reflecting reduced discriminatory power of individual items.

Item difficulty, defined as the proportion of correct responses, indicated a moderate range of difficulty across items in both assessments. Overall, the final examination contained slightly easier items than the baseline exam, suggesting a shift in the distribution of item difficulty between measurement points. Only the pre-test contained one “difficult” item and none contained “very difficult” items.

### Experience with the CBCT oral radiology platform

#### Impact and motivation

**Fig. 6:**
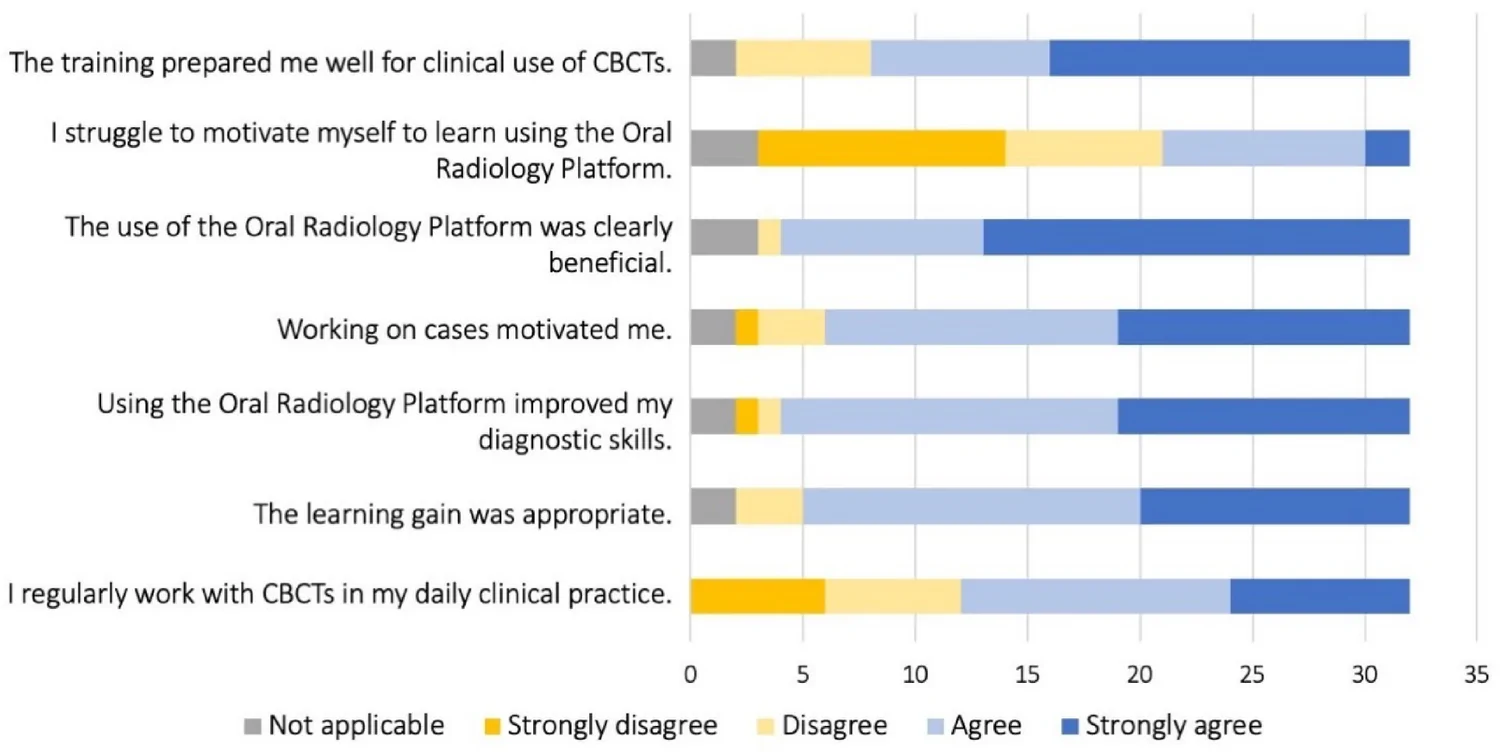
Impact and motivation. Bar graph illustrating the distribution of responses, independent of CBCT license status (*n* = 32)

Overall, the ORP was perceived as useful for enhancing clinical skills in the context of CBCT interpretation (Fig. 6). Dentists reported that the platform effectively improved their diagnostic abilities and was highly motivating, with the majority expressing satisfaction with the interactive case-based approach.

Detailed responses from the two groups (with and without CBCT licenses) are provided in Suppl. Fig. 1. The p-values from Fisher's exact test are provided in Suppl. Table 1, indicating no statistically significant differences between the groups for any of the items. However, dentists with the CBCT license tended to use CBCTs more regularly in their daily practice than those without (*p* = 0.07).

#### Usability and structure

**Fig. 7:**
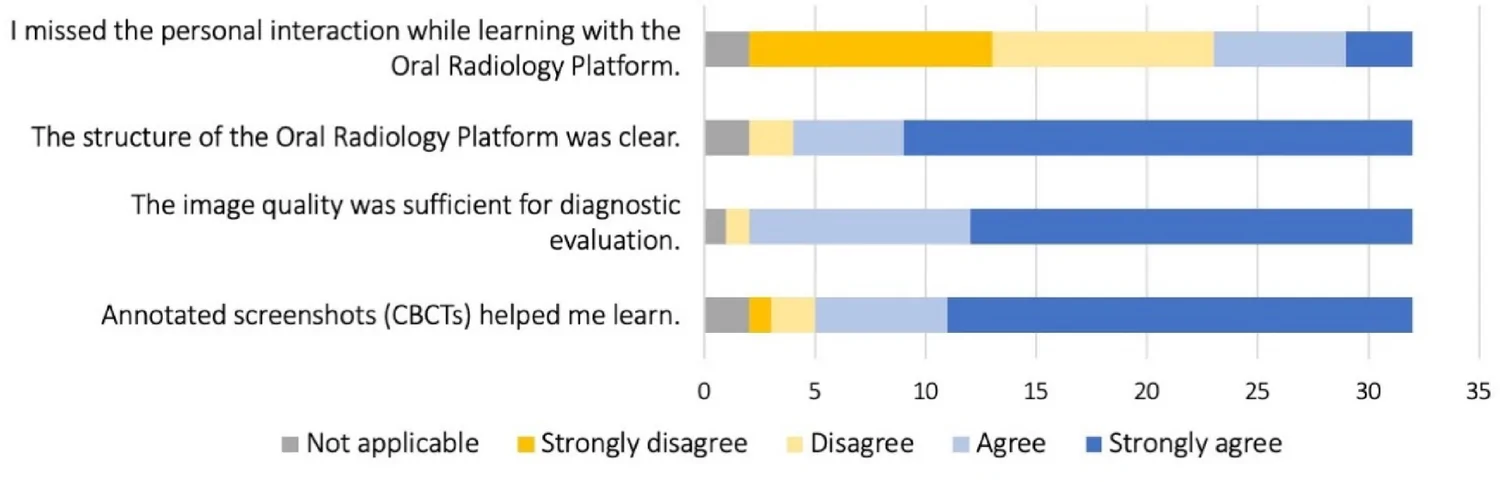
Usability and structure. Bar graph illustrating the distribution of responses, independent of CBCT license status (*n* = 32)

Overall, participants reported not to have missed personal interaction while using the ORP for learning, and more than 50% of respondents strongly agreed that the platform's key features effectively contributed to their learning experience (Fig. 7). Specifically, they considered the annotated CBCT screenshots a valuable learning resource, found the image quality sufficient for diagnostic use, and appreciated the intuitive and well-organized structure of the platform.

Detailed responses from the groups with and without CBCT license are provided in Suppl. Fig. 2. The* p*-values from Fisher's exact test are presented in Suppl. Table 2, demonstrating no statistically significant differences between the groups for any of the items.

## Discussion

Access to the annotated 2D CBCT Oral Radiology Platform enhanced the interpretation of the CBCT cases and CBCT-based knowledge among the German dentists participating in the study. The observed 9.3% increase in pre- to post-test scores reflects a clear and statistically significant improvement, highlighting the benefits of the digital platform. Although participants with a CBCT license achieved higher scores, the differences between the groups were no longer significant after the learning period, indicating that the platform effectively helped to close the initial difference in baseline test performance.

Consistent with previous studies, online interactive platforms have demonstrated high potential to enhance radiology education for dentists [11, 12, 20]. This finding is consistent with reports from the European Academy of DentoMaxilloFacial Radiology [10], which highlight that many dentists receive limited, if any, formal training in CBCT interpretation during undergraduate education.

The present study aligns with and extends the observations of previous work from our group focusing on 2D radiographs [14], demonstrating that an interactive ORP can not only effectively enhance 2D skills, but also CBCT interpretation for licensed dentists. Mücke et al. [14] reported improved undergraduate performance with the ORP during pandemic-related online-only learning. Our study shows that integrating annotated CBCT screenshots derived from 3D datasets leads to statistically significant knowledge gains for graduated dentists.

Participants with a CBCT license had higher baseline scores (75.8%) than those without a license (69.3%), reflecting their greater familiarity and prior experience with CBCT imaging in clinical practice. Notably, after the 8–10 week learning period, the difference between the groups was no longer statistically significant, suggesting that the platform effectively helped to close the initial difference in baseline performance (83.1% vs. 80.6%). Consequently, the platform offers valuable learning opportunities for both experienced clinicians and those without formal CBCT license, potentially supporting ongoing professional development in oral radiology.

The period of 8 to 10 weeks of access to the platform provided participants with the opportunity to use it daily on a variety of devices, including smartphones and tablets. Access via mobile devices allows learners to integrate platform use into their everyday routine, offering benefits for learning as previously noted [21]. It should be emphasized that continuous learning is essential for building foundational knowledge and for maintaining interpretation and diagnostic skills over time [22]. The extension of this learning period was chosen partly to roughly reflect a typical academic semester in dental school and partly because of German regulations for obtaining the German CBCT license, which require a similar learning period of about 3 months [9]. This also reflects that the learning was done alongside their normal clinical work and everyday professional life. Future studies without time restrictions might show whether using the platform for a longer period could improve long-term knowledge retention.

These results underscore the potential of the platform for ongoing professional development. In the future, the digital platform could provide continuous learning opportunities, enabling clinicians to regularly refresh and update their CBCT knowledge. It would be of interest to evaluate the effectiveness not only of annotated CBCT screenshots but also of annotated interactive 3D datasets, further enhancing its educational value and offering users more comprehensive CBCT training. The platform and participant pool were limited to German-speaking users. However, an international version could allow users to switch between languages, facilitating the exchange of knowledge and experience among dentists worldwide. In this context, it would be interesting to explore whether the present results are consistent across different countries, where CBCT licensing requirements and educational and training structures may differ.

As demonstrated by Lee et al. [12], digital learning platforms such as ours are widely accepted by users and can support learning in dental radiology. It should be emphasised that subjective perceptions of everyday clinical experience, self-reported motivation to use the platform, and perceived clinical relevance of CBCT were also included in the evaluation.

Annotated 2D datasets derived from CBCT volumes considered by the participants to be valuable tools for teaching and communicating 3D content. The absence of statistically significant differences between the two groups suggests comparable learning experiences among participants with and without prior CBCT-specific training. This lack of statistically significant difference was also observed in the study by Lee et al. [12], where satisfaction ratings on a 5-point scale showed no significant variation in relation to previous clinical experience (< 2, > 2, and > 5 years).

A limitation of the present platform is the absence of interactive 3D reconstruction and free volumetric navigation. Participants evaluated standardized 2D screenshots derived directly from CBCT software, preserving the original image quality and multiplanar views while limiting user-controlled orientation of the dataset. As a consequence, the educational implications of this restriction remain unclear. However, in clinical practice, CBCT interpretation is largely slice-based, with diagnostic decisions typically made on selected 2D levels [23]. Therefore, annotated 2D screenshots still capture essential cognitive steps of real-world CBCT evaluation and likely facilitate the transfer of learning to practical 3D interpretation. The study does not control for test–retest effects, exposure bias, or general learning over time, which limits the ability to attribute performance improvements solely to the platform.

Current evidence on 3D visualization in dental CBCT education is scarce [24, 25]. While previous studies have explored the role of 3D reconstruction in the assessment of specific anatomical structures [25], the present platform exposed participants to a broad spectrum of clinically relevant CBCT findings and pathologies encountered in daily practice. Future developments may incorporate interactive 3D visualization, volumetric rendering, or augmented-reality technologies, which have shown educational potential in other medical disciplines [26–28], to determine whether these features provide additional benefits beyond structured case-based learning.

The interpretation of the observed improvement is limited by the psychometric properties of the assessment instrument. Both the baseline and final questionnaires demonstrated questionable to poor internal consistency (Cronbach’s alpha = 0.66 and 0.54, respectively), with reduced item discrimination in the final test, contributing to its lower overall reliability. As the questionnaire was developed for this pilot study and was not formally validated, construct validity cannot be fully established. A predefined item pool was used, with items assigned alternately and consecutively to the pre- and post-test, with one question per assessment; however, no formal equivalence testing between the resulting test forms was performed, limiting the ability to exclude differences in test characteristics. The lower reliability and weaker discrimination observed in the post-test might reflect these differences, which could have influenced the magnitude of the observed improvement beyond actual learning gain. Therefore, the findings should be interpreted with caution, and future research should focus on formal validation of the instrument to ensure its psychometric robustness.

Overall, this study demonstrates that digital learning formats are becoming increasingly relevant for teaching 3D radiology skills, especially for dentists already working in clinical practice who seek to easily and conveniently enhance their knowledge on portable devices.

## Conclusions

Within the limitations of this pilot study, access to the structured and annotated CBCT learning platform improved the radiological knowledge of the participating dentists, regardless of whether they held a German CBCT license. The initial difference in baseline knowledge between participants with and without a formal CBCT license was successfully bridged and both groups demonstrated comparable learning gain following platform use. Nearly 90% of participants reported a perceived increase in knowledge. The platform was rated positively with regard to usability, clarity, and overall user satisfaction, with perceived beneficial impact on the clinical use of CBCT.

## Supplementary Information

Below is the link to the electronic supplementary material.

Supplementary Material 1

Supplementary Material 2

Supplementary Material 3

## Data Availability

Data are available upon reasonable request from the corresponding author.

## Abbreviations

2D: Two-dimensional
3D: Three-dimensional
95% CI: 95% Confidence interval
CBCT: Cone-beam computed tomography
IQR: Interquartile range
SD: Standard deviation
