# Supplementary material for: Impact of an e-learning platform in improving dentists’ CBCT diagnostic skills: a pilot study
Source: Oral Radiol. 2026 Jul 24;42(4):1144–53. doi: 10.1007/s11282-026-00954-2 (PMC13619758; doi:10.1007/s11282-026-00954-2)
Supplement: Supplementary file 1 [file 11282_2026_954_MOESM1_ESM.docx]

**SUPPLEMENTARY MATERIAL**

**TITLE**

Impact of an E-Learning Platform in Improving Dentists’ CBCT Diagnostic Skills: a Pilot Study.

**JOURNAL NAME** Oral Radiology

**AUTHORS**

Daniel Brockes^1^, Caroline V. Busch^1,2^*, Kathrin Becker^3^, Jürgen Becker^1^, Giulia Brunello^1,3^, Katharina Mücke^3,4^

**AFFILIATIONS**

^1^ Department of Oral Surgery, University Hospital Düsseldorf, Moorenstr. 5, 40225 Düsseldorf, Germany; daniel.brockes@med.uni-duesseldorf.de (D.B.); uniprofessorbecker@googlemail.com (J.B.); giulia.brunello@med.uni-duesseldorf.de (G.B.)

^2^ Department of Oral Medicine, Infection, and Immunity, Harvard School of Dental Medicine, Boston, MA, USA

^3^ Charité ‐ Universitätsmedizin, Freie Universität Berlin and Humboldt‐Universität zu Berlin, Department of Orthodontics and Dentofacial Orthopaedics, Aßmannshauser Str. 4-6, 14197 Berlin, Germany; kathrin.becker@charite.de (K.B.); katharina-maria.muecke@charite.de (K.M.)

^4^ Department of Orthodontics, University Hospital Düsseldorf, Moorenstr. 5, 40225 Düsseldorf, Germany

* corresponding author:	Dr. Caroline V. Busch

Department of Oral Surgery

University Hospital Düsseldorf

Moorenstr. 5 - 40225 Düsseldorf (Germany)

E-mail: caroline.busch@med.uni-duesseldorf.de

***Suppl. Figure 1.*** *Impact and motivation. Bar graph illustrating the distribution of responses in dentists with and without CBCT license (n=16 per group).*

***Suppl. Table 1.*** *Impact and motivation. P-values from Fisher's exact test were used to assess the influence of the CBCT license on platform evaluations. In the first analysis, "Strongly agree" and "Agree" were combined, as were "Disagree" and "Strongly disagree," with "Not applicable" excluded. A second analysis included "Not applicable" as a separate category.*

| **Item** | **1^st^ analysis (2 groups)** | | **2^nd^ analysis (3 groups)** | |
| --- | --- | --- | --- | --- |
|  | **No. responses included in the analysis** | **P-value** | **No. responses included in the analysis** | **P-value** |
| The training prepared me well for clinical use of CBCTs. | n = 30 | 0.65 | n = 32 | 0.82 |
| I struggle to motivate myself to learn using the Oral Radiology Platform. | n = 29 | 1.00 | n = 32 | 1.00 |
| The use of the Oral Radiology Platform was clearly beneficial. | n = 29 | 1.00 | n = 32 | 1.00 |
| Working on cases motivated me. | n = 30 | 0.60 | n = 32 | 0.79 |
| Using the Oral Radiology Platform improved my diagnostic skills. | n = 30 | 1.00 | n = 32 | 1.00 |
| The learning gain was appropriate. | n = 30 | 1.00 | n = 32 | 1.00 |
| I regularly work with CBCTs in my daily clinical practice. | n = 32 | 0.07 | n = 32 | 0.07 |

***Suppl. Figure 2.*** *Usability and structure. Bar graph illustrating the distribution of responses in dentists with and without CBCT license (n=16 per group).*

***Suppl. Table 2****. Usability and structure. P values from Fisher's exact test used to assess the influence of the CBCT license on platform evaluations. In the first analysis, "Strongly agree" and "Agree" were combined, as were "Disagree" and "Strongly disagree," with "Not applicable" excluded. A second analysis included "Not applicable" as a separate category.*

| **Item** | **1^st^ analysis (2 groups)** | | **2^nd^ analysis (3 groups)** | |
| --- | --- | --- | --- | --- |
|  | **N responses included in the analysis** | **P-value** | **N responses included in the analysis** | **P-value** |
| I missed the personal interaction while learning with the Oral Radiology Platform. | n = 30 | 0.43 | n = 32 | 0.70 |
| The structure of the Oral Radiology Platform was clear. | n = 30 | 1.00 | n = 32 | 1.00 |
| The image quality was sufficient for diagnostic evaluation. | n = 31 | 0.48 | n = 32 | 0.48 |
| Annotated screenshots (CBCTs) helped me learn. | n = 30 | 1.00 | n = 32 | 1.00 |
