# Supplementary material for: Impact of an e-learning platform in improving dentists’ CBCT diagnostic skills: a pilot study
Source: Oral Radiol. 2026 Jul 24;42(4):1144–53. doi: 10.1007/s11282-026-00954-2 (PMC13619758; doi:10.1007/s11282-026-00954-2)
Supplement: Supplementary file 2 [file 11282_2026_954_MOESM2_ESM.docx]

**APPENDIX 1 – Representative questions translated into English**

**Multiple-choice question**

A colleague shows you a screenshot of a CBCT scan. Please assess the scans, taking into account any endodontic, implantological and pathological abnormalities. Which of the following statement(s) is/(are) most likely to be correct?

**a)** Teeth 11, 22 and 13 have undergone root canal treatment, whilst teeth 21 and 12 appear untreated.

**b)** Tooth 15 shows a root filling with overfilled endodontic material apically.

**c)** In the region of the apex of tooth 21, there is a distinct, sharply defined apical translucency, consistent with periapical infection*.*

**d)** Tooth 23 shows a homogeneous root filling without any apical abnormalities.

**e)** In region 24, a dental implant is visible in the axial CBCT image.

**Single-choice question**

Examine the two-dimensional CBCT image for signs of inflammatory reactions and overfilled root canal filling material at tooth 16. Which of the following statement(s) is/ (are) most likely to be correct?

L

R

**a)** The left maxillary sinus presents no pathological findings.

**b)** The root filling of tooth 16 looks homogenies and there is no periapical change; the endodontic treatment is adequate.

**c)** The CBCT findings suggest a root-tip resection on tooth 16, accompanied by periapical radiolucency, which indicates chronic inflammation in the region of the buccal root.

**d)** In addition to the inflammatory lesion on the vestibular root of tooth 16, overfilling is also evident on the palatal root.

**e)** Tooth 46 shows a horizontal fracture in the area of the cementoenamel junction, which is associated with vertical bone loss in the furcation area.

**Kprim question**

A 61-year-old female patient presents with vague symptoms in the left upper jaw. She experiences occasional pain at pressure. A CBCT scan is performed for further investigation.

Please assess the statements below.

|  | **Correct** | **Incorrect** |
| --- | --- | --- |
| **a)** The bone substitute material placed in the edentulous region 25/26 shows a cortical border of the sinus; inflammatory involvement of the maxillary sinus can therefore be ruled out. | *○* | ○ |
| **b)** The scan reveals a correctly inserted implant in region 26 which, from a functional perspective, can be considered suitable for prosthetic loading. | *○* | ○ |
| **c)** An irregular opacity is visible in the left maxillary sinus, which is consistent with a polypoid formation or mucosal thickening and, in terms of differential diagnosis, may be of inflammatory or reactive origin. | *○* | ○ |
| **d)** In the right maxilla, two neighbouring implants show a reduced inter-implant distance; this may lead to reduced peri-implant bone stability in the long term. | *○* | ○ |
